# “By Hook or by Crook” - Meaning in Life for Older Adults Residing in Medicaid-Funded Assisted Living Facilities

**DOI:** 10.1093/geroni/igaf122.1722

**Published:** 2025-12-31

**Authors:** Brianna Morgan, Kelseanne Breder, Daniel David

**Affiliations:** New York University Langone Health, New York, New York, United States; New York University, New York, New York, United States; New York University, New York, New York, United States

## Abstract

Meaning in life (MiL) is the comprehension of one’s life and its purpose and is associated with well-being. Wong (2021) characterizes four categories of MiL: Enjoyment, responsible action, purpose, and understanding. Older adults residing in Medicaid-funded assisted living programs (ALP) have unique experiences that may influence how they describe MiL. This qualitative descriptive study aims to characterize MiL for older adults residing in ALP, illuminating their psychosocial needs. We applied a five-phase qualitative data analysis process from Bingham et al., (2023) to semi-structured interviews with older adults. We used trustworthiness methods of co-coding, peer debriefing, and community advisor board review. Older adults described experiences across all four MiL categories with cross-cutting themes: 1. Remaining connected 2. Feeling (in)dependent 3. Engaging with (in)justice 4. Keeping the faith Second, structural and contextual factors, such as systemic racism and ageism, financial and housing instability, and lack of trust and privacy influenced how participants experienced MiL. These findings highlight that older adults residing in ALP may experience structural and contextual challenges, such as inconsistent access to resources. Yet, they have robust MiL experiences characterized by meaningful connections, fierce independence, engagement with justice, and strong faith. These results inform policy and practice in ALF settings. Adapting individual-level meaning-making interventions using participatory co-design methods can incorporate the unique needs of ALP residents. System-level programs should facilitate connection, independence, engagement in justice, and faith while addressing contextual barriers. Overall, engaging older adults with how they experience MiL has the potential to enhance well-being for all.

